# Progression of stroke deficits in patients presenting with mild symptoms: The underlying etiology determines outcome

**DOI:** 10.1371/journal.pone.0231448

**Published:** 2020-04-24

**Authors:** Naveed Akhtar, Saadat Kamran, Hisham Elkhider, Soha Al-Makki, Noha Mhjob, Lubna ElShiekh, Hassan AlHussain, Musab Ali, Rola Khodair, Faisal Wadiwala, Abdul Salam, Dirk Deleu, Reny Francis, Ashfaq Shuaib

**Affiliations:** 1 The Neuroscience Institute, Hamad Medical Corporation, Doha, Qatar; 2 Department of Medicine, Stroke Program, University of Alberta, Edmonton, Canada; University of Ioannina School of Medicine, GREECE

## Abstract

**Background and purpose:**

Patients with acute stroke and mild or rapidly improving symptoms frequently show progression. The role of reperfusion treatment in such patients is not clear. We hypothesized that progression was most likely in patients with cortical localization and such patients may benefit from thrombolysis.

**Material and methods:**

We interrogated Hamad Stroke Database to evaluate 90-days outcome in patients with acute ischemic stroke admitted within 4 hours and a NIHSS score of ≤6. Evaluation was based on localization (lacunar or cortical), multi-model imaging abnormalities and whether they received rt-PA. The 90-day mRS was used to determine outcome.

**Results:**

During study period 6381 patients were admitted with acute stroke. Mild stroke within 4 hours was diagnosed in 506 [no thrombolysis: 381(lacunar: 213; cortical: 168), thrombolysis: 125 (lacunar: 45; cortical: 80)]. The rt-PA treated patients had significantly higher NIHSS (2.94±3.9 versus 1.28±2.46, p<0.0001), increased rates of complications (16.0% versus 3.9%, p<0.0001) and longer hospital stay (6.05±8.1 versus 3.78±3.6 days; p<0.001). In patients with cortical stroke, intracranial arterial occlusions (11.6% vs 3.9%, p<0.0001) and CTP mismatch (22.2% vs 4.4%, p<0.0001) were more frequent in rt-PA treated patients. Discharge mRS (33.6% versus 13.9%, p<0.001) and 90-days mRS (23.2% versus 11.8%, p = 0.002) was significantly worse in patients with cortical stroke (rt-PA-treated and untreated patients).

**Conclusions:**

The outcome in patients with mild stroke depends on lesion location (lacunar versus cortical) and severity of symptoms. Patients who receive rt-PA have significantly larger deficits, increased imaging abnormalities and higher rates of hospital complication, explaining the poor outcome in such subjects.

## Introduction

Uncertainty persists in the use of reperfusion therapy in patients with mild or rapidly improving symptoms following acute stroke (AS). [1,2] Reports suggest that 29–43% of patients do not get tissue plasminogen activator (r-tPA) solely because of mild symptoms. [3–5] This is particularly disconcerting as significant proportion may show progression of symptoms during hospitalization and require long-term care. [1–4] While the recently published PRISMS study failed to show benefits with the use of r-tPA in patients with mild stroke, [6] it did not achieve its desired recruitment and the majority of patients enrolled had subcortical lacunar strokes. Furthermore, two recently published meta-analysis also failed to provide any useful information in patients with mild strokes. [7,8]. There are however recent data that patients with cortical stroke [9] and with higher NIHSS score (4–5 vs. 1–3) may be more likely to show progression. [10]

The definitions of “mild stroke” and “rapidly improving symptoms” are unfortunately not very clear. [11] It is often left to the discretion of the neurologist [3,5] or low scores (0–6) on the National Institute of Health Stroke Scale (NIHSS). [12–14] The mechanisms of mild stroke include occlusion of an intra-cranial or extra-cranial artery or a sub-cortical ‘lacunar’ occlusion of a small vessel may differ from patients with rapidly improving symptoms and putting them together may not always be appropriate. [1] There is no clarity on the best medical management of such patients presenting acutely to the emergency department (1–3). Furthermore, most reports comprise of small number of patients [4,5,15,16] or had outcomes reported at discharge from hospital, [3,17] likely too early to estimate the true extent of disability. Additionally, there is often minimal imaging information available to explain the mechanism for the stroke. This is especially important if intracranial arterial occlusion is suspected where progression may be more likely [1,2] Most recently published meta-analysis also provides contradictory data on the efficacy of thrombolysis in patients with mild symptoms. [18,19,20,21] The first aim of our study was to evaluate if progression was more likely seen with cortical strokes, especially in the presence of intracranial arterial occlusion compared to sub-cortical lacunar-type’ stroke. Secondly, we aimed to compare the effectiveness of thrombolysis in patients with cortical vs. sub-cortical lacunar strokes.

## Methods

**Fig 1:**
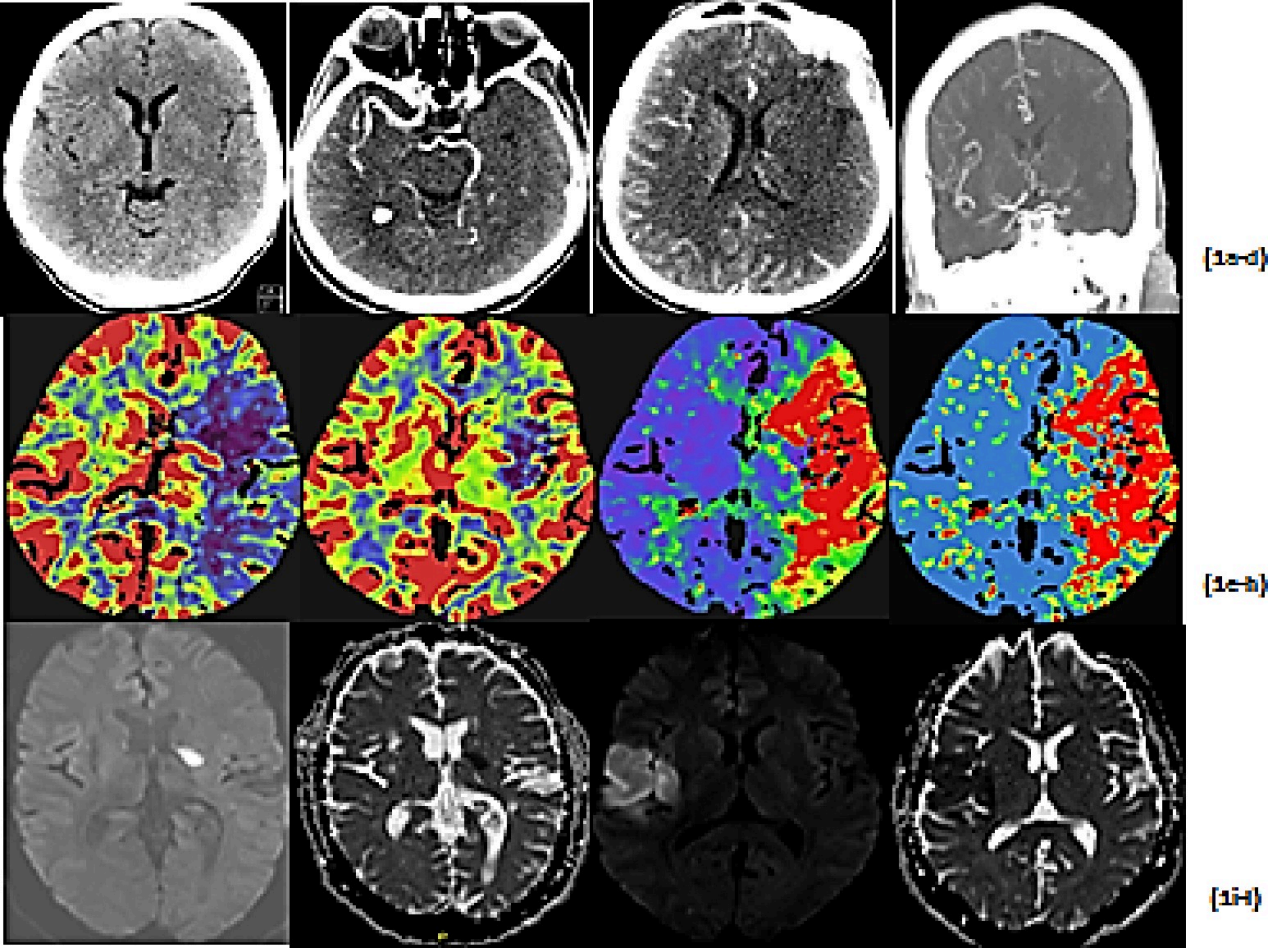
Localisation of stroke type based on imaging, (a) showing non-contrast CT brain, (b) CT angiogram showing occluded left Middle cerebral artery, (c) and (d) paucity of collateral circulation left MCA vessel, with (e) showing reduced cerebral blood flow in defined territory, (f) cerebral blood volume, (g) increased time to drain, and (h) increased mean transit time, (i) and (j) showing DWI and ADC map showing Lacunar stroke, and (k) and (l) showing DWI and ADC scan of a Cortical Stroke.

We analyzed data of patients admitted with a diagnosis of stroke or TIA to Hamad General Hospital (HGH) in Qatar from January 1, 2014 through April 2017 was analysed from a hospital based prospective stroke database. The study was approved by the Hamad Medical Corporation’s Institutional Review Board. HGH annually admits approximately 1800 patients with AS and approximately 160 patients are treated with rt-PA yearly [22, 23]. Stroke localization was made using the Trial of Org 10172 in AS Treatment (TOAST) criteria. [24] For the present analysis, patients admitted within 4 hours of symptom onset were reviewed in two categories; intra-cranial embolic or thrombotic large vessel disease, especially if imaging showed cortical involvement (cortical stroke) and small vessel disease when the acute lesion was within the deep structures (lacunar stroke) as defined by the clinical symptoms and localization on CT and MR imaging (Fig 1). The 4-hour time was used as the ‘cut-off’ because of the minimum ‘30 minutes’ time from admission to potential treatment with rt-PA. [25] The admission National Institute of Health Stroke Score (NIHSS) was used for selection of patients for further analysis. The outcome of patients treated and not treated with rt-PA was separately compared in patients with cortical and lacunar stroke as we hypothesized that progression was more likely to occur with cortical strokes. For ‘progression’ we used a clinical subjective deterioration as noted by the treating neurologist. Outcome was analyzed at discharge and 90. A modified Rankin Score (mRS) of 0–2 at 90-day was considered a good outcome and 3–6 as unfavorable outcome. The data was anonymized prior to analysis. The IRB did not consider this sufficient and did not require individual patient consent.

### Statistical analysis

All statistical analyses were performed using Statistical Package for Social Sciences Version-22 (SPSS). The distribution of continuous variables was assessed before using statistical tools. Descriptive results for all quantitative variables are presented as mean± standard deviation or median with inter-quartile range. Numbers (percentage) were reported for all qualitative variables. Mean level comparisons between patients among four different groups (Lacunar with rt-PA, Lacunar without rt-PA, cortical with rt-PA, and cortical without rt-PA) were assessed using ANOVA test and multiple comparison analysis was performed using Scheffe test. If assumption of an ANOVA test was violated then an alternative non-parametric Kruskal Wallis test was performed. Pearson Chi-Square test or Fisher Exact test was used to compare the proportion of all categorical variables among above mentioned four groups. Bi-variate analysis was also performed using Independent sample t-test or Mann Whitney U-test whenever appropriate to compare all quantitative variables between those received rt-PA versus those who didn’t receive rt-PA. Qualitative variables between two groups (rt-PA vs. no rt-PA) were compared using the Pearson Chi-square test or Fisher exact test as appropriate. Change in the mRS (0–2 vs. 3–6) from discharge to 90-days was compared using McNemar test. P value <0.05 (two-tailed) was considered statistically significant.

## Results

**Fig 2:**
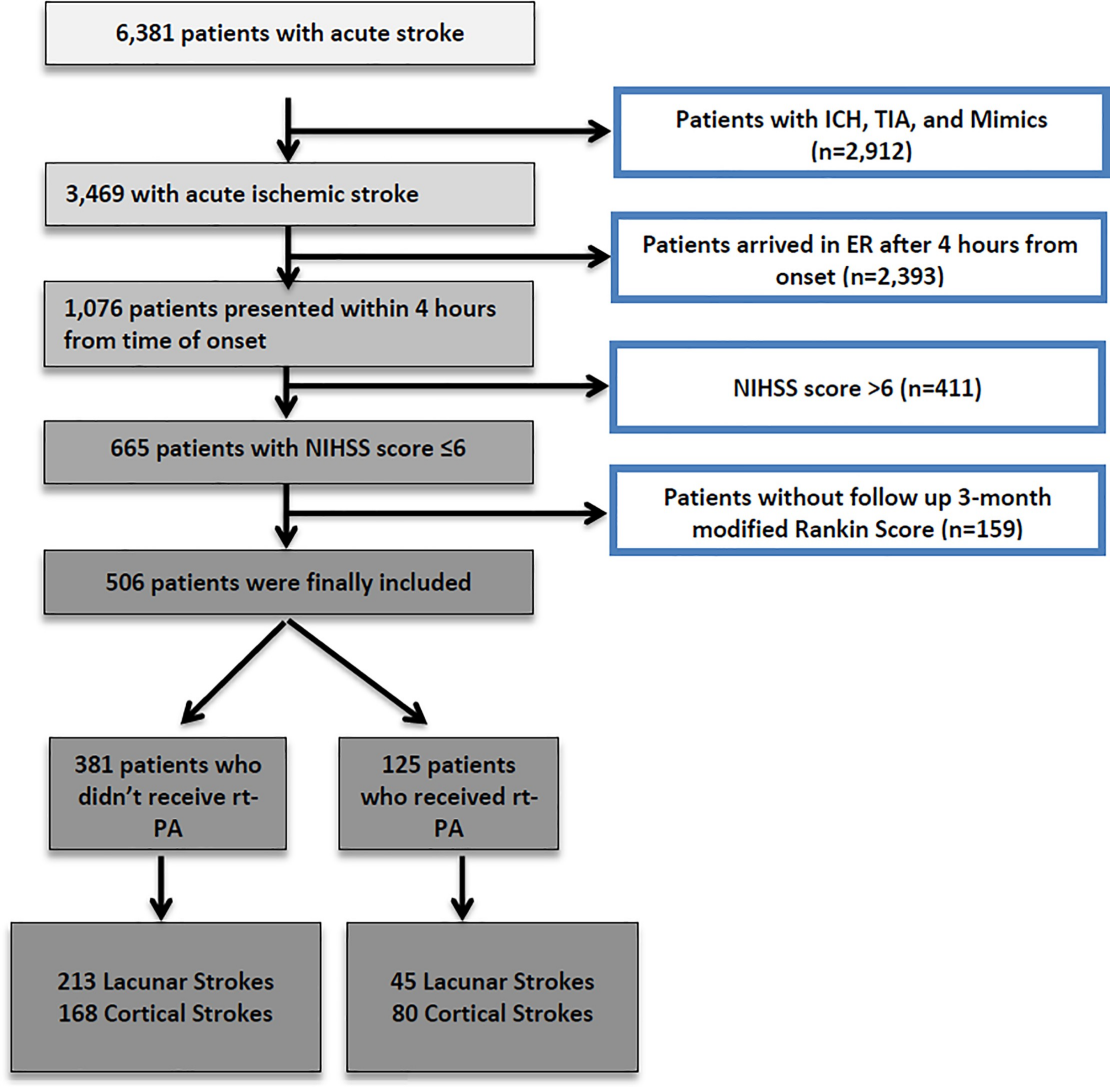
Selection of eligible patients.

During the study period, 6381 patients were admitted with a diagnosis of AS or TIA. After excluding TIAs, stroke mimics and intracerebral hemorrhage, 3469 patients with ischemic stroke were available for further analysis. There were 1076 (31.0%) patients who presented within 4 hours of symptoms-onset with 665 with an NIHSS of ≤6. Patients in whom the 90-day mRS was not available (159 patients) were excluded from further analysis. A total of 506 patients were included in the final analysis. Amongst these patients with mild symptoms, 381 patients who did not receive rt-PA (lacunar 213; cortical 168) and 125 patients were treated with rt-PA (lacunar 45; cortical 80) (Fig 2, Table 1).

**Table 1:** Demographics and clinical features of patients with Lacunar Stroke (LS), Cortical Stroke (CS), LS with rt-PA and CS tPA having NIH SS ≤6 and arrived to ED ≤4 hours.

	Total Patients with NIHSS < 6, ONSET < = 4hr (n = 506)	Patients with NIHSS < 6, ONSET < = 4hr, No rt-PA, and SVD (n = 213)	Patients with NIHSS < 6, ONSET < = 4hr, No rt-PA, and LVD (n = 168)	Patients with NIHSS < 6, rt-PA given, and SVD (n = 45)	Patients with NIHSS < 6, rt-PA given, and LVD (n = 80)	P- Value
**Age**	54.27±11.9	54.83±11.1	55.15±12.9	53.20±13.1	51.54±11.3	0.128
**Male**	435 (86.0)	180 (84.5)	141 (83.9)	42 (93.3)	72 (90.0)	0.255
**DM on admission (n = 366)**	195 (53.3)	82 (52.6)	59 (51.8)	23 (60.5)	31 (53.4)	0.816
**HTN on admission (n = 366)**	252 (68.9)	104 (66.7)	81 (71.1)	26 (68.4)	41 (70.7)	0.873
**Dyslipidemia on admission (n = 366)**	193 (52.7)	79 (50.6)	59 (51.8)	24 (63.2)	31 (53.4)	0.575
**Prior Stroke**	53 (10.5)	24 (11.3)	25 (14.9)	1 (2.2)	3 (3.8)	0.013
**Prior TIA**	5 (1.0)	2 (0.9)	3 (1.8)	0	0	0.504
**Prior CAD**	67 (13.2)	23 (10.8)	33 (19.6)	5 (11.1)	6 (7.5)	0.023
**AF on admission (n = 364)**	36 (9.9)	3 (1.9)	21 (18.8)	0	12 (20.7)	0.0001
**Prior Smoking (n = 366)**	97 (26.5)	38 (24.4)	30 (26.3)	9 (23.7)	20 (34.5)	0.490
**CTA done**	301 (59.5)	97 (45.5)	91 (54.2)	38 (84.4)	75 (93.8)	0.0001
**CTP done**	279 (55.1)	92 (43.2)	76 (45.2)	38 (84.4)	73 (91.3)	0.0001
**Door to CT Brain (minutes)**	92.62±145.8	115.79±169.3	103.91±155.4	40.87±51.62	37.90±27.85	0.0001
**Vascular occlusion Present (n = 312)**	36 (11.5)	0	19 (19.8)	0	17 (22.4)	0.0001
**Perfusion Deficit Present (n = 297)**	45 (15.2)	0	16 (18.4)	0	29 (38.7)	0.0001
**Mean NIHSS on Admission**	2.65±1.9	2.01±1.5	2.07±1.8	4.40±1.3	4.60±1.3	0.017
**Mean NIHSS at Discharge**	1.84±3.1	1.26±1.9	1.31±2.9	2.97±3.2	2.92±4.2	0.0001
**Length of Stay**	4.35±5.2	3.07±2.9	4.69±4.1	3.92±3.3	7.25±9.6	0.0001
**Mortality at 90 days**	11 (2.2)	1 (0.5)	8 (4.8)	0	2 (2.5)	0.026
**Recurrent Stroke**						
**Complications**	35	1 (0.5)	14 (8.3)	3 (6.7)	17 (21.3)	0.0001
**Cholesterol levels**	4.87±1.2	4.86±1.1	4.64±1.2	5.43±1.5	5.06±1.2	0.055

Results are expressed as mean ± standard deviation, and number (percentage).

* P-value was calculated to compare all the predictors among patients with NIHSS < 6, ONSET < = 4hr and small vessel disease (with or without r-tPA) and Cortical Stroke (with or without r-tPA) groups.

LS = Lacunar Stroke, Cortical Stroke (CS), DM = Diabetes Mellitus, HTN = Hypertension, TIA = Transient ischemic Attack, CAD = Coronary artery disease, AF+ Atrial Fibrillation, CTA = CT Angiogram, CTP = CT Perfusion, NIHSS+ National Institute of Stroke Scale

### Outcome in patients with mild stroke and in whom rt-PA was not used

The mean NIHSS on admission for the entire group was 2.04±1.64. The NIHSS at discharge of patients with no rt-PA was significantly lower than patients treated with rt-PA (1.28±2.46 versus 2.94±3.9, p <0.0001). The most common reasons for not treating patients with rt-PA are shown in Table 2. Symptoms too mild or rapidly improving were the most common reason for not offering thrombolysis.

**Table 2:** Reasons for No thrombolysis in patients with Lacunar Stroke (LS), Cortical Stroke (CS) who presented with acute stroke within 4 hours.

REASONS	Total Patients (n = 381)	LS Patients with No rt-PA (n = 213)	CS Patients with, No rt-PA (n = 114)
**Out of Window**	**2 (0.5)**	**1 (0.5)**	**1 (0.6)**
**Low NIHSS**	**176 (46.2)**	**104 (48.8)**	**72 (42.9)**
**Improved Symptoms**	**136 (35.7)**	**86 (40.4)**	**50 (29.8)**
**Established Infarction on CT**	**27 (7.1)**	**10 (4.7)**	**17 (10.1)**
**Late Referral or Missed Diagnosis**	**3 (0.8)**	**0**	**3 (1.8)**
**Refused by Patient/Family**	**2 (0.5)**	**1 (0.5)**	**1 (0.6)**
**Intracerebral Bleed on CT**	**7 (1.8)**	**4 (1.9)**	**3 (1.8)**
**Contraindications/Trauma**	**28 (7.3)**	**7 (3.3)**	**21 (12.5)**

Results are expressed as mean ± standard deviation, and number (percentage).

LS = Lacunar Stroke, Cortical Stroke (CS).

**Fig 3:**
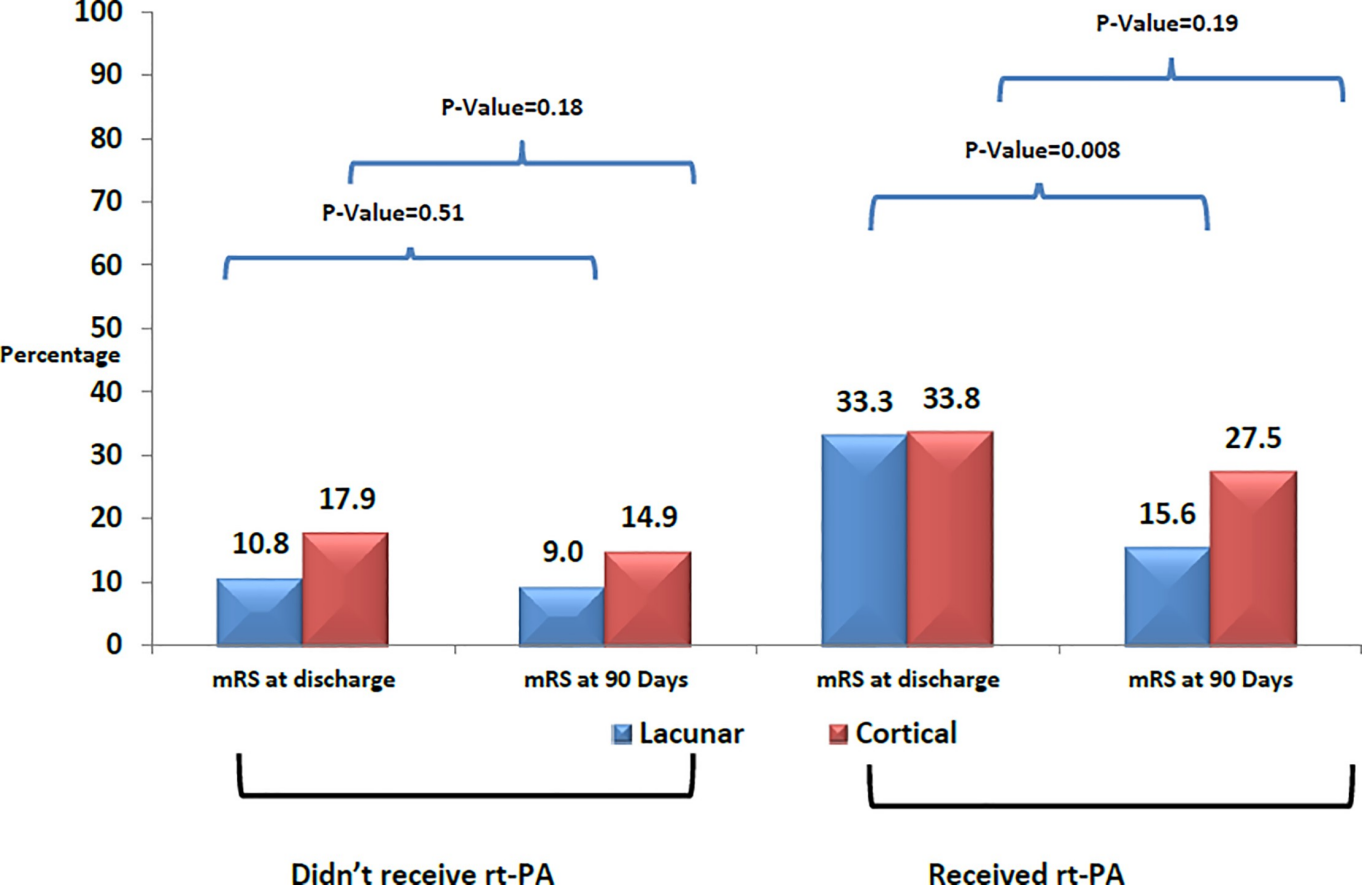
Poor outcome (modified Rankin Score 3–6) at discharge and at 90 days for patients with lacunar, cortical, lacunar with rt-PA and cortical with rt-PA (no rt-PA- n = 381, rt-PA- n = 125).

The discharge and 90-day clinical outcome using mRS (0–2 versus 3–6) for patients with cortical and subcortical lacunar stroke is shown in Fig 3. Compared to cortical strokes, there were significantly fewer patients with a poor outcome with lacunar strokes. There was no significant improvement in 90-day evaluation compared to discharge in both cortical (p = 0.18) and in lacunar strokes (p = 0.51)

### Outcome with mild stroke where t-PA was offered to the patients

Mean Door-to-CT times were significantly shorter in patients treated with rt-PA (38.97± 37.9 versus 110.65 ± 163.3 minutes, p< 0.0001). Patients with cortical stroke treated with r-tPA had significantly higher NIHSS (cortical rt-PA: 4.60±1.3 versus no rt-PA: 4.40±1.3; P = 0.017). Patient who received rt-PA had significantly more complications (16.0% versus 3.9%, p<0.001), longer length of stay in hospital (mean 6.05±8.1 versus 3.78±3.6) (p<0.001) and had an unfavourable outcome at discharge and at 90 days when compared to patients in whom rt-PA was not used (Fig 3). In patients treated with rt-PA, lacunar stroke showed significantly better improvement during the time between discharge and 90-days (66.6% to 84.4%, p = 0.008) compared to patients with cortical stroke (66.2% vs 72.5%, p = 0.19, Fig 3).

**Fig 4:**
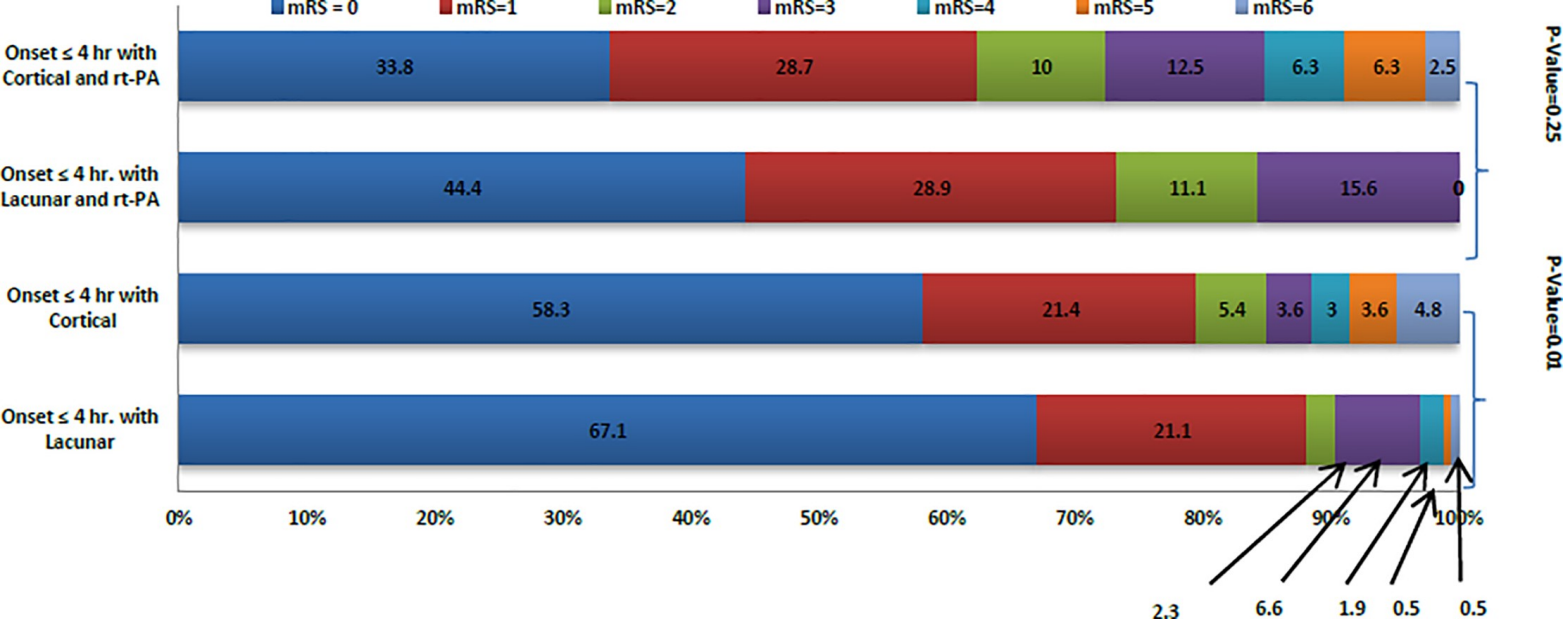
Inter and intra-group comparison of outcome at discharge and at 90-days.

There were significant differences in outcome when patients treated with rt-PA were compared to patients not thrombolysed. Poor outcome at discharge (33.6% versus 13.9%, p<0.001) and 90-days (23.2% versus 11.8%, p = 0.002) was worse in patients treated with rt-PA compared to no treatment. Fig 4 shows inter and intra-group comparison of outcome at discharge and at 90-days.

### Multimodal cranial imaging

All patients had cranial CT scans at admission. Multi-model CT (CTA and CTP) were done in 43.6% (166/381) and cranial MRI (DWI, SWI, T1W, T2W, FLAIR), and MRA brain and post-contrast MRA neck was completed within 2 days of admission in 72.9% (278/381) patients. The main reason for not doing CTP was a clinical diagnosis of small vessel (lacunar) stroke. The major reason for not completed MRI included, early discharge of the patient from hospital, claustrophobia or medical reasons (for example; pacemaker, metals in body). Six patients that were originally classified as lacunar small vessel disease were categorized into the ‘cortical’ category once the imaging studies were completed. More patients treated with rt-PA had CTA and CTP (88.0% versus 43.6%, p≤0.0001). The number of patients with cortical stroke with CTP mismatch was significantly higher in rt-PA treated patients (29/75 (38.7% versus 16/87 (18.4%), p = 0.004). Intracranial arterial occlusions, mostly in the distal M1 or M2 branch were seen in only 17/76 patients (22.4%).

## Discussion

This is a single center series with the following observations. Patients with mild stroke symptoms require multimodal CT or MR imaging in order to separate patients with cortical involvement from lacunar stroke. Mild lacunar stroke does not require thrombolysis and improve spontaneously in most cases. In patients with cortical stroke, worsening may occur, especially in the presence of intracranial arterial occlusion or perfusion mismatch. Our observations are similar to the CATCH study where the presence of intracranial occlusion was associated with worse outcome in patients with TIAs or mild stroke. [25] The worse outcome in our study in patients with cortical stroke who received r-tPA was likely secondary to several factors including, more severe stroke, higher rates of imaging abnormalities and higher frequency of medical complications. The higher NIHSS score likely led to the decision for thrombolysis.

The exact definition of ‘mild stroke’ is problematic. In earlier studies [3, 15–17] and in the ‘Get with the guidelines’ (GWTG) registry, [4,26] the definition was left to the discretion of neurologist. In other studies, where NIHSS was used to define “mild stroke”, the cut-off varied between “4–6”, [14,24,27] although most studies reported a cut-off of “5”. [24,28] A score of < 2 or patients with isolated symptoms (gaze palsy, dysarthria or facial weakness) has also been suggested to define mild stroke. [7,29] In general, stroke severity and long-term functional outcome is related to NIHSS [30] but the outcome is not always linear. It is not uncommon to see significant functional disability even when the NIHSS has returned to zero. [1]

Despite multiple reports, there is uncertainty in determining outcome in patients with mild symptoms. [1,2] Some studies show poor outcome, [3,17] while other show that the outcome may be more benign. [14, 31] In addition, the practice of lumping together patients with mild symptoms and those with rapidly improving symptoms may not be appropriate as they often may have dissimilar etiologies and most likely different outcomes. [1,25,32–33] A recent publication from GWTG registry showed short-term outcome better for patients with rapidly improving symptoms compared to those with stable mild symptoms. [30] Similar to our study, patients with higher NIHSS were significantly more likely to receive rt-PA and 90-days outcome was also related to the admission NIHSS. In our series, admission NIHSS had a major influence on the decision to thrombolysis and long-term outcome. Other studies also showed that milder stroke patients with higher admission NIHSS are more likely to have a poorer short-term outcome. [7–8,26,34] In the largest series from GWTG (93,517 patients) presenting within 2 hours from onset, 26% did not receive rt-PA solely because they had mild or rapidly improving symptoms. [13] At discharge 28% could not be sent home. The admission NIHSS was between 1–5. The risk of poor outcome increased with increasing NIHSS scores. Patients discharged home had lower NIHSS, were younger and had fewer vascular risk factors. [13]

A major strength of our research is in classifying mild stroke patients according to their mechanism. The early and frequent use of multi-model imaging is useful in identifying stroke mechanism and predicting patients likely to worsen following IS [35] or may benefit from thrombolysis. [36] A limitation of our study is that multi-model imaging was completed in approximately 50% of patients. Our work is similar to the report of Ali et al. [26] In their series, older patients with higher NIHSS, larger stroke size on imaging and poor collaterals were more likely to show progression. [26] Katari et al also reported that early worsening was most frequently seen in patients when early infarction growth was evident on MRI. [37] A study from UCLA also showed deterioration often evident in patients with intracranial vascular occlusion. Progression was seen in 3% with no occlusion compared to 38% of patients with occlusion. [27] Similarly, Nedeltchev et al also identified a higher NIHSS and vessel occlusion to be associated with poor outcome. [8] Finally in the CATCH study, intracranial occlusion was associated with a 19% early deterioration compared to 2% in patients with no occlusion. [38]

The evidence for the use of rt-PA in patients with acute mild stroke is unclear. In most studies patients were evaluated in the thrombolysis time window of between 3 hours [3] and 6 hours. [28] A few studies have looked at outcome in patients presenting up to 24 hours. [34] The NINDS trial and IST-III study analyzed their data on thrombolysis to placebo in patients with mild strokes. In NINDS trial 52 patients with NIHSS of 0–5 were randomized (42 = rt-PA and 16 = placebo). Minimal or no disability was seen in 78.6% of rt-PA treated and 81% of placebo-treated patients on mRS assessment at 90 days. [24] Similarly in the IST-III, there were no significant differences in patients treated with rt-PA or placebo with mild symptoms and within 3 hours from onset. [28] A recent meta-analysis of patients treated with rt-PA also included subjects with mild strokes (NIHSS 0–4). There was a significant 10% treatment effect with thrombolysis. [18] In our study, 96 patients were treated with rt-PA in the 4.5 hour window. Higher admission NIHSS and frequent imaging abnormalities were the only significant factors associated with increased rt-PA use. The outcome of patients treated with rt-PA, especially with cortical stroke was significantly worse than patients in whom rt-PA was not offered. This likely reflects the more severe stroke in treated group and higher frequency of abnormalities on multi-model imaging. This may also be related to the somewhat slower response times in treatment in patients with AS with mild symptoms. [39]). The PRISMS randomized clinical trial evaluated the usefulness of rt-PA in mild stroke [6]. Unfortunately, the study did not reach its required number of patients 948 due to slow enrollment. In a comparison of 157 rt-PA treated patients to 156 placebo-treated patients, there was no benefits in the active treatment arm [6] The current on-going trials will be helpful in a better understanding of the role of thrombolysis or thrombectomy in patients with mild or rapidly improving symptoms (NCT02072226; TASTE, TEMPO-2 and ACTRN 12613000243718).

There are several other strengths in this study. This is the largest single center study of mild strokes where multi-model imaging was used to determine the etiology and pathophysiology of the acute event. We compared the outcome in patients with two major stroke mechanisms. Our data shows that patients with small vessel lacunar stroke have a better outcome and may not require reperfusion therapy. We also show that patients with cortical strokes related to large vessel disease or cardio-embolic stroke are more prone to progression, especially in the presence of intracranial vascular occlusion or perfusion deficits. Finally, we were able to show that the admission NIHSS is the most important determinant of the use of rt-PA in patients with mild stroke.

There are a few limitations to our study. This is not a randomized comparison of the use of rt-PA in patients with mild stroke. It is possible that some patients in the lacunar category may have an embolic mechanism but these are likely to be very few. The poor outcome in rt-PA treated patients is difficult to explain but is likely related to the more severe stroke. We also realise that the higher rates of mild stroke in our population is related to the poorly controlled hypertension and diabetes in this population [23] and may not be reflective of the population in North America or Europe.

## Conclusion

We report our experience with AS and mild symptoms in a large database. Most patients with lacunar stroke make a full recovery compared to cortical stroke, especially where imaging identifies intracranial vascular occlusion or diffusion abnormalities. We also describe that the use of rt-PA increases as the admission NIHSS increase. Classification of patients into small vessel disease or cortical stroke is helpful in identification of outcome in patients with AS and mild symptoms.
